# The gut microbiome and metabolome in children with a first febrile urinary tract infection: a pilot study

**DOI:** 10.1007/s00467-025-06782-6

**Published:** 2025-05-14

**Authors:** Barbora Piteková, Ivan Hric, Jakub Zieg, Eva Baranovičová, Patrik Konopásek, Jakub Gécz, Paul J. Planet, Viktor Bielik

**Affiliations:** 1 Department of Pediatric Emergency Medicine, National Institute of Children’s Diseases, Bratislava, Slovakia; 2 Department of Pediatric Urology, Faculty of Medicine, Comenius University and National Institute of Children’s Diseases, Bratislava, Slovakia; 3 Department of Pediatrics, Slovak Medical University in Bratislava, Bratislava, Slovakia; 4 Biomedical Center, Institute of Clinical and Translational Research, Slovak Academy of Sciences, Bratislava, Slovakia; 5 Department of Biological and Medical Sciences, Faculty of Physical Education and Sport, Comenius University, Bratislava, Slovakia; 6 Department of Pediatrics, Second Faculty of Medicine, Charles University, Prague, Czech Republic; 7 Biomedical Center Martin, Jessenius Faculty of Medicine in Martin, Comenius University in Bratislava, Martin, Slovakia; 8 Department of Children and Adolescents, Third Faculty of Medicine, Charles University and University Hospital Kralovske Vinohrady, Prague, Czech Republic; 9 Children’s Hospital of Philadelphia, Philadelphia, PA USA; 10 Department of Pediatrics, University of Pennsylvania, Philadelphia, PA USA

**Keywords:** Gut microbiota, SCFA, Kidney, Pyelonephritis, Pediatric

## Abstract

**Background:**

Urinary tract infection (UTI) is a common bacterial infection in the pediatric population. Febrile urinary tract infection (fUTI) can lead to severe complications such as urosepsis as well as kidney scarring, chronic kidney disease, and systemic hypertension. Recent research supports the hypothesis that dysbiosis of the microbiome may play a role in the pathogenesis and development of fUTI in infants. Our main aim was to compare the shift in gut microbiota composition between children with the first fUTI and controls.

**Methods:**

We conducted an observational study with 17 children with the first fUTI compared to 18 healthy controls. We performed analysis of the gastrointestinal microbiome and measurements of metabolites in stool and urine.

**Results:**

In the gut microbiome, we found significant differences with lower α-diversity the Shannon index) and significantly lower relative abundance of probiogenic bacteria (short-chain fatty acids (SCFA)) in children with the first episode of fUTI before the start of antibiotic therapy. Furthermore, our findings confirm that the length of breastfeeding has significant influence on gut microbiota composition, reducing pathogenic bacteria and enhancing beneficial taxa. Shannon diversity, duration of breastfeeding, and specific taxa, particularly *Faecalibacterium* and *Escherichia*, emerged as strong predictors linked to the development of fUTI.

**Conclusions:**

This study demonstrates that gut microbiome changes are associated with the onset of fUTI in children. Machine learning models identified Shannon index, specific bacterial taxa, and breastfeeding as strong predictors of fUTI. The study highlighted the potential role of the gut microbiome in preventing fUTI.

**Graphical abstract:**

**Figure:**
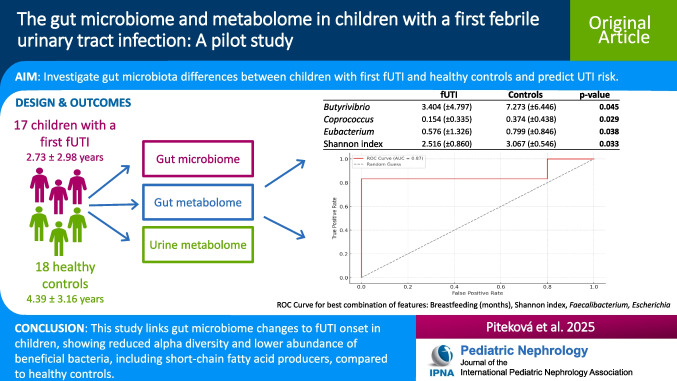
A higher resolution version of the Graphical abstract is available as Supplementary information

**Supplementary Information:**

The online version contains supplementary material available at 10.1007/s00467-025-06782-6.

## Introduction

Acute urinary tract infection (UTI) is a common bacterial infection in children. If not diagnosed or treated promptly, febrile UTI (fUTI) may cause acute complications such as urosepsis as well as long-term consequences such as kidney scarring which can lead to hypertension and kidney function impairment over time [1]. Uropathogenic *Escherichia coli* (UPEC), which is thought to originate in the gut microbiome, is the most common etiology of fUTI possessing virulence factors that enable strong adhesion to the urinary tract epithelium [2]. The gastrointestinal microbiome consists of billions of bacteria, viruses, fungi, and other microorganisms and has important functions in digestion, metabolism, protection against pathogens, and regulation of the immune system [3]. Shifts in the intestinal microbiome have been associated with several diseases, including allergies, chronic inflammatory bowel diseases, autism, diabetes mellitus, and others [4, 5]. Furthermore, recent research supports the hypothesis that dysbiosis of the microbiome plays a critical role in the development of fUTI in infants [6–8]. Children with fUTI have significant changes in gut microbiota [7]. The gut is the main reservoir for UTI pathogens, and it has been suggested that disturbances of the gut microbiome (e.g., high prevalence of pathogenic *Escherichia coli*) lead to the development of fUTI [6, 9]. Moreover, the gut microbiota has an important role in regulating the immune system [3, 10]. Dysregulation of immune balance may promote the translocation of uropathogens from the gut to the urinary tract and increase the susceptibility to developing fUTI [6–8, 10]. However, it is not clear if the gut microbiota changes precede the onset of fUTI and potentially predict its incidence.

Therefore, the purpose of this observational study was to investigate differences in the gut microbiota between children with a first attack of fUTI and those without history of fUTI to better understand the role of the gut microbiome in the development of fUTI. This study also aims to predict UTI based on microbial features and a history of breastfeeding, which may offer valuable insights into the role of the microbiome in UTI susceptibility.

## Methods

### Sample collection

Children aged 28 days to 17 years + 365 days (*n* = 17) experiencing their first episode of fUTI [fever in their medical history, elevated inflammatory parameters: C-reactive protein (CRP) higher than 20 mg/l, presence of pyuria (> 15 elements/µl urine), and significant bacteriuria, determined by cultivation from mid-stream urine sample as a detection of uropathogens responsible for UTI (count ≥ 10^5^ colonies/mL)] who were examined at the National Institute of Children’s Disease at the Department of Pediatric Emergency Medicine in Slovakia from April 1, 2022 to August 31, 2023 were included in this prospective case control study. Every included child provided a stool sample before the start of antibiotic therapy. Stool samples were stored in a stabilization solution (deoxyribonucleic acid (DNA)/ribonucleic acid (RNA) shield fecal collection tubes) and kept at a temperature of − 80 °C until laboratory analyses. During the examination at the Department of Pediatric Emergency Medicine, every child provided a mid-stream urine sample before the start of antibiotics, and microbial culture was performed.

Children with recurrent UTI, insignificant bacterial count in urine culture, or those with stool samples that were taken after the beginning of antibiotic treatment were excluded. No child had a known CAKUT (congenital anomalies of the kidney and urinary tract) or other urinary tract malformation prior to the development of fUTI, and none had any symptoms of bladder or bowel dysfunction. The males included in the study were not circumcised. As a control group, healthy children, with no history of UTI or kidney disease, without known chronic diseases, and without fever or any signs of acute infection at the time of sample collection, were included (*n* = 18). Inclusion in the study required signed informed consent from a legal guardian. Basic characteristics of patients were obtained from medical records. Generally, we excluded every child who had received antibiotics in the 3 months preceding the measurements. The goal was to minimize the confounding effects of antibiotic-induced disruption of the microbiota. Both short- and long-term antibiotic treatments have been shown to alter gut microbial composition, with recovery periods ranging from several weeks to months, depending on the treatment duration and individual factors [11, 12].

### Personal history

Information about the delivery mode and duration of breastfeeding was obtained from the parents of every child enrolled in the study.

### Microbial analysis

Fecal DNA was purified using the ZymoBIOMICS DNA/RNA kit (Zymo Research, USA) following the manufacturer’s instructions. Next-generation sequencing (NGS) libraries were prepared using the 16S Microbiome NGS Assay (ViennaLab, Austria). The V3-V4 regions were amplified in the first polymerase chain reaction (PCR), followed by a second PCR with dual index sequences for sample demultiplexing. PCR products were cleaned using Agencourt AMPure XP magnetic beads (Beckman Coulter, USA). DNA libraries were quantified with a Qubit 2.0 Fluorometer and verified using an Agilent 2100 Bioanalyzer. Libraries were diluted to 4 nM, and sequenced on the Illumina MiSeq platform with 300-bp paired-end reads.

### Illumina data processing

The sequencing data were analyzed using software included in the ViennaLab NGS Microbiome Assay kit. Read preprocessing was performed with BBMerge, Cutadapt, and SeqKit [13, 14]. Species-level classification of reads was conducted using the CLARK classification system [15], which relies on discriminative k-mers within a sequence database. The sequence databases employed in the classification pipeline were constructed using data from the SILVA [16] and UNITE [17, 18] databases. Taxonomic information for read classification was obtained from the National Center for Biotechnology Information (NCBI) taxonomy database (https://www.ncbi.nlm.nih.gov/taxonomy/). Diversity metrics were derived from species-level abundance data utilizing MOTHUR software [19].

The summation (SUM) of butyrate producers includes the relative abundances of eight key genera (*Anaerostipes*, *Eubacterium*, *Coprococcus*, *Ruminococcus*, *Faecalibacterium*, *Butyrivibrio*, *Megasphaera*, and *Lachnospira*), which are recognized as major contributors to butyrate production in the gut [20]. In contrast, the SUM of pathogenic bacteria focuses on the relative abundances of genera with well-documented roles in pathogenicity, including *Escherichia*, *Enterobacter*, *Enterocloster*, *Enterococcus*, *Klebsiella*, and *Salmonella* [21, 22].

### Stool and urine metabolites concentrations; nuclear magnetic resonance (NMR)

#### Data acquisition

The concentrations of 21 metabolites were analyzed in the stool samples, which belong to the following groups: SCFA, amino acids, simple organic acids, and alcohols. The concentrations of the following 16 metabolites were assessed in the urine sample: acetone, acetate, pyruvate, succinate, dimethylamine, citrate, trimethylamine, carnitine, hippurate, hippurate quinolinate, histidine, 1-methylnicotineamide, hypoxantine, and hypoxantine formate. Concentrations of selected stool and urine metabolites were determined using nuclear magnetic resonance (NMR) analysis, as described by Baranovičová et al. [23].

#### Statistics

The statistical data analyses were conducted using Statistical Package for the Social Science (SPSS) 21.0 for Windows (SPSS, Inc., Chicago, IL, USA). The normality of the data was evaluated with the Shapiro–Wilk test. For parametric data (gut microbiome, gut, and urine metabolome), an independent *t* test was employed to compare the groups, while the Mann–Whitney *U* test was used for non-parametric data. Spearman’s correlation coefficient was calculated to assess the associations between variables. A significance level of *p* < 0.05 was considered statistically significant. For the calculation of the duration of breastfeeding and composition of microbiome, logistic regression was used.

Random Forest (RF) was used for classification based on decision trees. The models were trained using the RandomForest Classifier from the scikit-learn library [24]. The model's performance was evaluated using several metrics, including accuracy and the area under the curve (AUC), which measures its discriminatory power across different classification thresholds. The receiver operating curve (ROC) was also plotted to visualize the trade-off between sensitivity and specificity at various thresholds.

To understand the predictive contributions of individual variables, variable importance (VIMP) measures were calculated based on the reduction in prediction error when each variable was included in the model. Higher VIMP values indicated greater importance in the prediction process.

#### Ethics

The study was approved by the Ethics Committee of the National Institute of Children’s Diseases in Slovakia with the approval number EK/6/4/2022. The legal guardians of every patient enrolled in the study gave written consent to enroll in the study and signed informed consent.

## Results

### Demographic data

We prospectively enrolled 17 patients with fUTI and 18 controls. Generally, patients were 3.58 ± 3.10 years old. Characteristics of patients and healthy controls are recorded in Table 1. Urine cultures revealed *Escherichia coli* as the most common pathogen for the first fUTI in 94.11% (*n* 16/17). In just one case, *Klebsiella aerogenes* was isolated. All urine samples in the control group were sterile. No child had a known CAKUT or other urinary tract malformation prior to development of fUTI. Each child with an fUTI underwent kidney ultrasonography at the time of diagnosis. Kidney ultrasonography showed no abnormalities in 11 children with fUTI, while mild renal pelvis dilation unilaterally was found in six cases (SFU 1). All cases were classified as uncomplicated fUTI according to the latest guidelines of the American Academy of Pediatrics (AAP) [25].

**Table 1:** Demographic data of children enrolled in the study

	Patients with febrile UTI	Healthy controls	*p* value
**Age** (in years) Mean ± SD	2.73 ± 2.98 (*n* = 17)	4.39 ± 3.16 (*n* = 18)	0.1092
**Gender** (female/male)	58.82% vs. 41.17% (10/7, *n* = 17)	27.77% vs. 72.22% (5/13, *n* = 18)	0.130
BMI* (in kg/m^2^) Mean ± SD	15.3 ± 0.91 (*n* = 12*)	15.5 ± 1.22 (*n* = 15*)	0.156
**Breastfeeding** (in months) Mean ± SD	3.58 ± 2.45 (*n* = 17)	4.56 ± 1.95 (*n* = 18)	0.243
**C-section**	41.17% (*n* 7/17)	33.33% (*n* 6/18)	0.896

^*^BMI was calculated only in children older than 2 years

### Microbiome

We found a significant difference in the α-diversity (Shannon index) between the control group (3.056) and the fUTI group (2.051), *p* = 0.05. Principal component analysis revealed significant differences in the abundance of certain bacterial taxa and bacterial phyla between the control group and patients with fUTI. The known beneficial bacteria and butyrate-producers like Bacteroides were significantly lower in relative abundance in children with fUTI. The complete results are shown in Table 2.

**Table 2:** Proportion of bacterial taxa in the gut microbiome in children with a first episode of fUTI vs. healthy controls

	fUTI (*n* = 17)	Controls (*n* = 18)	*p* value
Taxa			
* Bacteroidetes*	10.008 (± 12.071)	16.546 (± 11.960)	**0.045**
* Bacteroidia*	9.877 (± 11.931)	16.368 (± 11.957)	**0.049**
* Oligoflexia*	0.003 (± 0.009)	0.051 (± 0.145)	**0.032**
* Bacteroidales*	9.871 (± 11.934)	16.367 (± 11.957)	**0.049**
* Eubacteriaceae*	0.578 (± 1.331)	0.803 (± 0.853)	**0.038**
* Agathobaculum*	0.094 (± 0.192)	0.345 (± 0.303)	**0.005**
* Butyrivibrio*	3.404 (± 4.797)	7.273 (± 6.446)	**0.045**
* Coprococcus*	0.154 (± 0.335)	0.374 (± 0.438)	**0.029**
* Dialister*	0.243 (± 0.515)	1.125 (± 1.528)	**0.017**
* Eubacterium*	0.576 (± 1.326)	0.799 (± 0.846)	**0.038**
* Lucifera*	0.006 (± 0.026)	0.201 (± 0.303)	**0.02**
* Sutterella*	0.389 (± 1.582)	0.198 (± 0.505)	**0.049**
Species			
* Agathobaculum desmolans*	0.094 (± 0.192)	0.345 (± 0.303)	**0.005**
* Bacteroides galacturonicus*	0.006 (± 0.014)	0.032 (± 0.061)	**0.045**
* Blautia obeum*	0.137 (± 0.182)	0.490 (± 0.736)	**0.045**
* Butyrivibrio fibrisolvens*	3.346 (± 4.751)	7.269 (± 6.443)	**0.045**
* Clostridium tertium*	0.067 (± 0.198)	0.427 (± 1.076)	**0.015**
* Coprococcus comes*	0.092 (± 0.164)	0.216 (± 0.214)	**0.027**
* Limosilactobacillus fermentum*	0.059 (± 0.089)	0.252 (± 0.268)	**0.007**
* Lucifera butyrica*	0.006 (± 0.026)	0.201 (± 0.303)	**0.02**
* Sutterella wadsworthensis*	0.384 (± 1.583)	0.195 (± 0.506)	**0.038**
Shannon index	2.516 (± 0.860)	3.067 (± 0.546)	**0.033**

### Metabolome

Using NMR data acquisition, we quantified metabolites from 26 classes of compounds in the urine and stool samples. Significant differences in metabolite concentrations were found in both urine and stool samples (see Tables 3 and 4). Moreover, we found a higher concentration of butyrate in the control group compared to the fUTI group, although this difference did not reach statistical significance (control 0.012 vs. fUTI 0.008, *p* = 0.080). On the other hand, we identified a significant positive correlation between stool butyrate and the abundance of certain bacterial taxa such as *Faecalibacterium prausnitzii* (*r* = 0.622; *p* = 0.000), *Butyrivibrio fibrisolvens* (*r* = 0.575; *p* = 0.000), and *Anaerostipes cacceae* (*r* = 0.417; *p* = 0.014).

**Table 3:** Urine metabolites in children with a first episode of fUTI vs. healthy controls (relative concentrations of metabolites)

URINE	fUTI (*n* = 17)	Controls (*n* = 18)	*p* value
Acetate	262.709 (± 422.700)	42.950 (± 64.786)	**0.003**
Acetone	841.084 (± 1781.154)	82.034 (± 238.972)	**0.001**
DMA (dimethylamine)	335.650 (± 144.785)	239.277 (± 133.332)	**0.032**
TMA (trimethylamine)	61.080 (± 38.491)	32.279 (± 19.945)	**0.016**
Quinolinate	7.121 (± 7.356)	2.459 (± 1.766)	**0.007**
Hypoxanthine	8.454 (± 5.834)	5.644 (± 6.272)	**0.042**

**Table 4:** Stool metabolites in children with a first episode of fUTI vs. healthy controls (relative concentrations of metabolites)

STOOL	fUTI (*n* = 17)	Controls (*n* = 18)	*p* value
Lactate	0.00520 (± 0.00266)	0.00231 (± 0.00037)	**0.001**
Leucine	0.02259 (± 0.01156)	0.03385 (± 0.01278)	**0.011**
Isoleucine	0.00358 (± 0.00203)	0.00534 (± 0.00204)	**0.011**
Valine	0.01113 (± 0.00577)	0.01741 (± 0.00672)	**0.006**
Alanine	0.01821 (± 0.00724)	0.02449 (± 0.00942)	**0.037**
Fumarate	0.00037 (± 0.00042)	0.00057 (± 0.00039)	**0.013**
Tryptophan	0.00014 (± 0.00010)	0.00006 (± 0.00005)	**0.034**

### Predictors of fUTI

**Fig. 1:**
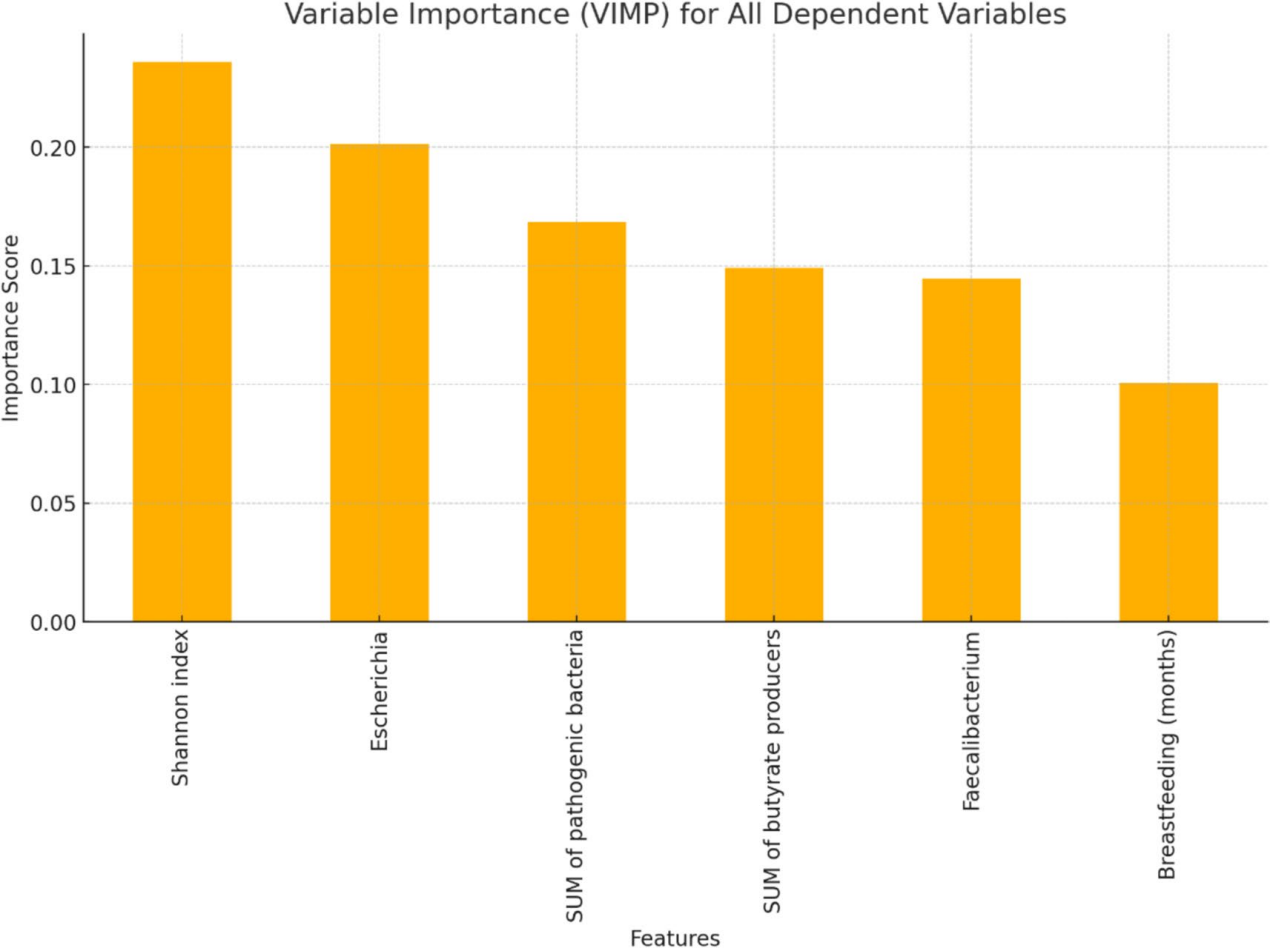
The VIMP graph depicts the contribution of each dependent variable to the prediction of UTIs based on a Random Forest model. The variables are ranked according to their relative importance in improving the model’s predictive accuracy. Features e.g., Shannon index, the relative abundance of *Escherichia*, and SUM of pathogenic bacteria demonstrate the highest importance, suggesting their significant role in distinguishing between cases with and without urinary infections. This analysis highlights the key variables influencing the prediction and their potential biological relevance

The Random Forest model was utilized to evaluate the predictive importance of all dependent variables in determining UTI status. The VIMP plot revealed that the Shannon index had the highest importance score, followed by the relative abundance of *Escherichia*, SUM of pathogenic bacteria, and SUM of butyrate-producing bacteria. Additionally, the relative abundance of *Faecalibacterium* and the breastfeeding (months) variable also contributed significantly to the model’s accuracy (Fig. 1). These features likely are critical factors associated with UTIs.

**Fig. 2:**
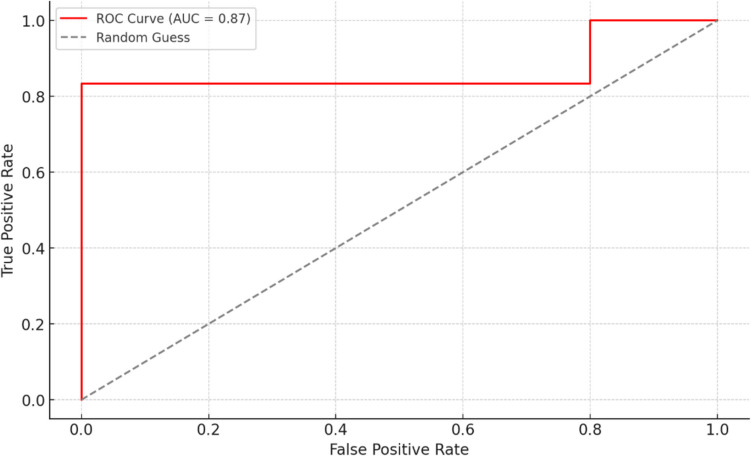
The ROC curve demonstrates the classification performance of the logistic regression model based on the optimal combination of four variables (joint predictors): breastfeeding (months), Shannon index, and the relative abundance of *Faecalibacterium* and *Escherichia*. The ROC curve assesses the model’s ability to distinguish between cases with and without UTIs. The AUC value of 0.87 indicates a strong predictive capability of the selected features. The diagonal dashed line represents the performance of a random classifier, serving as a baseline comparison. The model shows significant improvement over random guessing, highlighting the importance of the chosen variables in predicting urinary infection outcomes

The ROC analysis was conducted to evaluate the classification performance of logistic regression models for predicting UTI status based on selected features. By selecting specific features (length of breastfeeding, Shannon index, and abundance of selected bacteria such as *Faecalibacterium* and *Escherichia*), the model’s performance achieved an AUC of 0.87 (Fig. 2). This combination of variables demonstrated a strong capability to distinguish between cases with and without UTIs, highlighting potential predictive importance of duration of breastfeeding.

**Fig. 3:**
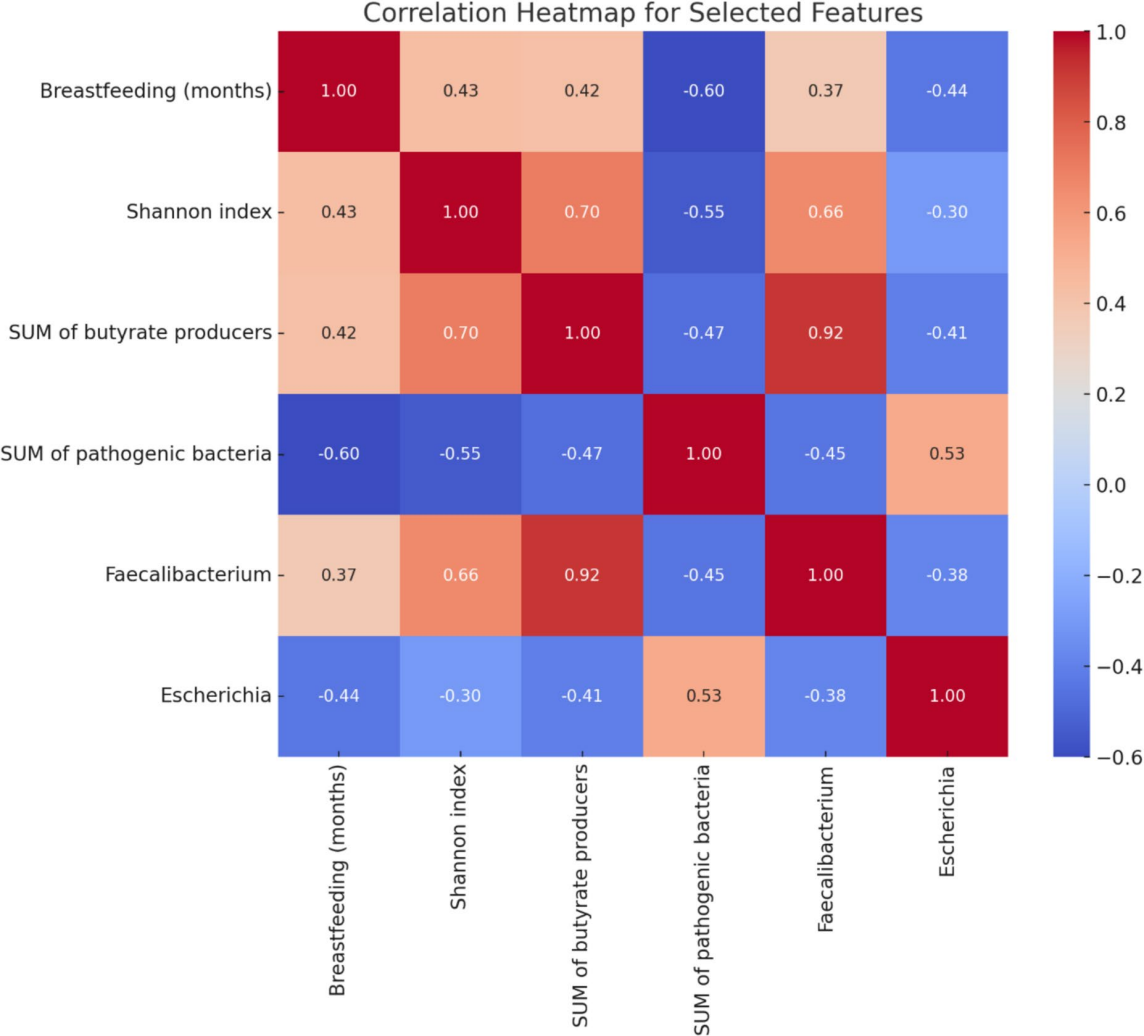
The pairwise correlations among the selected features (breastfeeding, Shannon index, SUM of butyrate producers, SUM of pathogenic bacteria, *Faecalibacterium*, and *Escherichia*). Positive correlations are represented in shades of red, while negative correlations are shown in shades of blue. The intensity of the color corresponds to the strength of the correlation, with values ranging from − 1 (perfect negative correlation) to + 1 (perfect positive correlation)

The correlation heatmap (Fig. 3) highlights the relationships among selected features, including length of breastfeeding, Shannon index, SUM of butyrate producers, SUM of pathogenic bacteria, *Faecalibacterium*, and *Escherichia*. The analysis revealed several noteworthy patterns. A strong positive correlation was observed between Shannon index and the SUM of butyrate producers (*r* = 0.70), suggesting that higher microbial diversity is associated with an increase in beneficial butyrate-producing bacteria. The SUM of pathogenic bacteria exhibited a moderate negative correlation with Shannon index (*r* = − 0.55), indicating that increased microbial diversity may be associated with a reduction in pathogenic bacterial abundance. The results show that high microbial diversity may play a protective role against UTIs.

## Discussion

In this observational study, we analyzed the gut microbiome and metabolome in children with the first episode of fUTI before the start of the antibiotic treatment. We aimed to discover whether there are existing changes in the gut microbiome in pediatric patients with the first episode of fUTI, which could significantly change the view of the pathogenesis of fUTI. We hypothesized that children with fUTI have a shift of gut microbiota alpha diversity and the abundance of *Escherichia coli* in the gut and stool. The main findings of our study were the significant differences in α-diversity (the Shannon index) and significantly lower relative abundance of probiogenic bacteria (SCFA) in children with fUTI. Furthermore, this study demonstrated the potential of machine learning models to predict UTIs based on microbial features, which may offer valuable insights into the role of the microbiome in UTI susceptibility. Bacterial genera such as *Faecalibacterium* and *Escherichia* emerged as key predictors in a model of logistic regression (Random Forest).

In the study conducted by Paalane et al. in 2018, the authors identified gut dysbiosis in children diagnosed with fUTI, including cases of the first episode as well as recurrent fUTI [7]. They sequenced the variable regions of the fecal bacterial *16S rRNA* gene in 37 children with fUTI and compared them with controls. At the genus level, they demonstrated a higher representation of *Enterobacteriaceae* and a lower representation of *Peptostreptoccocus* compared to controls [7].

In our study, we enrolled exclusively patients with the first episode of fUTI before antibiotic treatment. Our results demonstrate that changes in the microbiome are associated with the onset of fUTI. We did not observe higher representation of *Enterobacteriaceae* overall, but we did see an increase in *Escherichia*. We postulate that the first step in a cascade of the development of fUTI is a shift of gut microbiota alpha diversity and a reduction of probiogenic bacteria, that then leads to an increase in *Escherichia coli*. In cases of recurrent fUTI and repeated antibiotic administration, there is a progressive disruption of the gut microbiota, promoting the expansion of *Enterobacteria,* as reported by Paalane et al. [7]. To verify this hypothesis, further studies focusing on children with recurrent fUTI are needed.

Hong et al. recently analyzed the gut microbiome in preterm newborns [26]. They reported significantly higher abundance of UTI-causing pathogens (*Enterobacteriaceae* or *Enterococcaceae*) in infants with fUTI compared to those without fUTI. Although we did not include newborns, the results of this study supported our finding that changes in the gut are associated with the development of fUTI in children. Similarly, studies involving adult patients have reached comparable conclusions. Worby et al. studied adult women with recurrent non febrile UTI and observed significantly depleted microbial richness and butyrate-producing bacteria compared with controls [27]. These changes are like those observed in our study. These results support the theory of gut microbiome shift preceding the development of fUTI.

Magruder et al. observed in kidney transplant recipients, that relative gut abundance of *Escherichia* or *Enterococcus* was an independent risk factor for the development of chronic bacteriuria or UTI [28].

Furthermore, results of recent studies investigating the role of probiotics in the management of patients with recurrent fUTIs strengthened the connection between gut microbiota and fUTIs. Children who were treated with probiotics experienced longer interval without developing a new episode of fUTI [8].

It is known that factors such as mode of delivery, breastfeeding, antibiotic administration, and dietary habits contribute to changes in the gut microbiota [29]. Human milk contains probiogenic bacteria [30]. The role of breastfeeding in the prevention of fUTI in preterm children is well-established [31]. Our findings support the hypothesis that the length of breastfeeding has a significant impact on gut microbiota composition, reducing the abundance of pathogenic bacteria and enhancing the growth of beneficial taxa. This shift of the gut microbiome, in turn, could be associated with increased UTI risk.

The results of our study showed a strong association between changes in the gut microbiome and the development of fUTI. We found the alterations of gut microbiome in children with the first episode of fUTI. Moreover, our results show that the length of breastfeeding is a key early life modifiable determinant that influences microbiota composition and likely impacts the risk of development of fUTI. Knowledge of the link between gut microbiome and fUTI development may influence future new therapeutic approaches to prevent fUTI. In addition, it is important to prevent adverse changes in the microbiome by promoting breastfeeding, especially in children at risk of the development of fUTI at a later age.

Furthermore, we observed significant differences in urinary and fecal metabolites, which provide additional insights into the microbiome shifts associated with urinary infection [32, 33]. Specifically, we detected elevated levels of urinary acetate and dimethylamine, along with reduced concentrations of butyrate in stool samples of children with fUTI. These findings align with the decreased abundance of butyrate-producing bacteria, such as *Faecalibacterium* and *Butyrivibrio,* observed in the gut microbiome of affected children. Given the immunomodulatory role and inhibitory effect of SCFA on *Escherichia coli* growth [34], our results support the hypothesis that gut dysbiosis and its metabolic consequences may contribute to increased susceptibility to fUTI.

Our study has several limitations. First, the small sample size (*n* = 35) reduces the statistical power and generalizability of our findings. Additionally, while we observed differences in gut microbiota composition between children with fUTI and healthy controls, the lack of a temporal evaluation prevents us from determining whether these microbiome shifts precede fUTI or occur because of the infection. We also found a correlation between longer breastfeeding duration and a higher abundance of probiotic bacteria, suggesting a potential role in gut health. However, factors such as timing, exclusivity, and overall infant nutrition should be considered before drawing definitive conclusions.

A key strength of our study is that it provides novel insights into the association between gut microbiome composition and fUTI in a well-defined, homogeneous pediatric population before antibiotic treatment. Additionally, we used both microbiome and metabolome analyses, offering a comprehensive approach to understanding microbial and metabolic shifts in fUTI. Despite the small sample size, our findings highlight potential microbial predictors of fUTI, which can inform future larger-scale studies.

## Conclusions

This study demonstrates that gut microbiome changes are associated with the onset of fUTI in children. We observed decreased bacterial alpha diversity and reduced abundance of beneficial bacteria, such as short-chain fatty acid producers, in children with their first episode of fUTI. Machine learning models identified Shannon index, *Faecalibacterium*, *Escherichia*, and breastfeeding as joint predictors of fUTI. Overall, the study underscores the importance of gut microbiome health in preventing fUTI, with further research needed to explore therapeutic microbiome interventions.

## Supplementary Information

Below is the link to the electronic supplementary material.

ESM 1 Graphical abstract (PPTX 140 KB)

## Data Availability

All data generated or analyzed during this study are included in this published article.

## Abbreviations

AAP: The American Academy of Pediatrics
AUC: Area under the curve
CAKUT: Congenital anomalies of kidney and urinary tract
CRP: C-reactive protein
DMA: Dimethylamine
DNA: Deoxyribonucleic acid
fUTI: Febrile urinary tract infection
NCBI: National Center for Biotechnology Information
NGS: Next-generation sequencing
NMR: Nuclear magnetic resonance
PCR: Polymerase chain reaction
RF: Random Forest
RNA: Ribonucleic acid
ROC: Receiver operating curve
SCFA: Short-chain fatty acids
SPSS: Statistical Package for the Social Sciences
SUM: Summation
TMA: Trimethylamine
UPEC: Uropathogenic *Escherichia coli*
UTI: Urinary tract infection
VIMP: Variable importance
