# Supplementary material for: The gut microbiome and metabolome in children with a first febrile urinary tract infection: a pilot study
Source: Pediatr Nephrol. 2025 May 14;40(10):3145–54. doi: 10.1007/s00467-025-06782-6 (PMC12402013; doi:10.1007/s00467-025-06782-6)
Supplement: Supplementary file 1 — Graphical abstract (PPTX 140 KB) [file 467_2025_6782_MOESM1_ESM.pptx]

## Slide 1
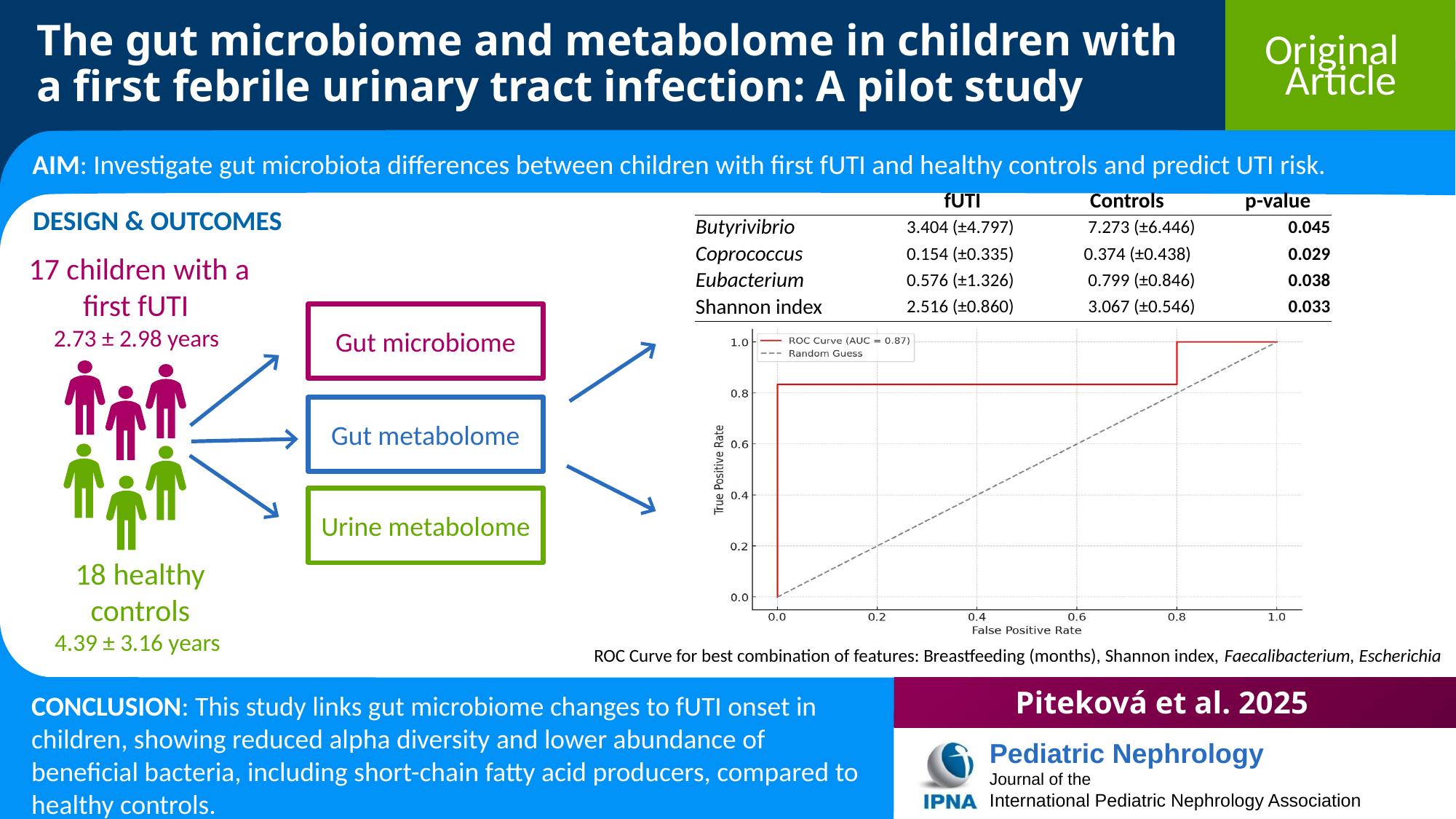

The gut microbiome and metabolome in children with a first febrile urinary tract infection: A pilot study
AIM: Investigate gut microbiota differences between children with first fUTI and healthy controls and predict UTI risk.
| | fUTI | Controls | p-value |
| --- | --- | --- | --- |
| Butyrivibrio | 3.404 (±4.797) | 7.273 (±6.446) | 0.045 |
| Coprococcus | 0.154 (±0.335) | 0.374 (±0.438) | 0.029 |
| Eubacterium | 0.576 (±1.326) | 0.799 (±0.846) | 0.038 |
| Shannon index | 2.516 (±0.860) | 3.067 (±0.546) | 0.033 |
DESIGN & OUTCOMES
17 children with a first fUTI
2.73 ± 2.98 years
Gut microbiome
Gut metabolome
Urine metabolome
18 healthy controls
4.39 ± 3.16 years
ROC Curve for best combination of features: Breastfeeding (months), Shannon index, Faecalibacterium, Escherichia
Piteková et al. 2025
CONCLUSION: This study links gut microbiome changes to fUTI onset in children, showing reduced alpha diversity and lower abundance of beneficial bacteria, including short-chain fatty acid producers, compared to healthy controls.
-----
